# Echinacoside exerts antidepressant-like effects through enhancing BDNF-CREB pathway and inhibiting neuroinflammation *via* regulating microglia M1/M2 polarization and JAK1/STAT3 pathway

**DOI:** 10.3389/fphar.2022.993483

**Published:** 2023-01-04

**Authors:** Renrui Lu, Li Zhang, Huihui Wang, Meng Li, Weisheng Feng, Xiaoke Zheng

**Affiliations:** ^1^ School of Pharmacy, Henan University of Chinese Medicine, Zhengzhou, China; ^2^ The Engineering and Technology Center for Chinese Medicine Development of Henan Province, Zhengzhou, China

**Keywords:** depression, echinacoside, M1/M2 polarization, neuroinflammation, JAK1/STAT3 pathway, BDNF

## Abstract

The present study was performed to investigate the antidepressant effect of echinacoside (ECH) using chronic unpredictable mild stress (CUMS) induced depression mice and lipopolysaccharide (LPS)-stimulated N9 microglial cells. CUMS treatment was performed on C57BL/6 mice for 28 days, followed by gavaging with different doses of echinacoside (15 and 60 mg/kg) for 21 consecutive days. Sucrose preference test (SPT), open field test (OFT), tail suspension test (TST), and forced swimming test (FST) were measured to assess the effects of echinacoside on CUMS-Induced Depressive-Like Behaviors. After that, the pathological changes of hippocampus were determined by Hematoxylin and eosin (HE) staining and Nissl staining; the neurotransmitters, pro-inflammatory cytokines and indoleamine 2,3-dioxygenase (IDO) levels, and the hypothalamic–pituitary–adrenal (HPA) axis activity were determined by enzyme linked immunosorbent assay (ELISA); Iba 1were evaluated by Immunofluorescence assay; Key protein expression levels of CREB/BDNF signal pathway were measured by western blotting. Subsequently, N9 cells were stimulated with 1 μg/ml LPS to induce N9 microglia activation, and were treated with 5–20 μM of echinacoside for 24 h. After that, the levels of NO, interleukin (IL)-1β, IL-6, tumor necrosis factor alpha (TNF-α), IL-4, IL-10, and transforming growth factor beta (TGF-β) in N9 cell culture supernatants were measured by enzyme-linked immunosorbent assay (ELISA) kits; morphology and Iba 1 expression level were observed by high-content screening assay; the M1 markers of CD11b, CD86 and M2 markers of CD206 were analyzed by imaging flow cytometry. Results show that treatment with echinacoside reversed CUMS-increased immobility time in OFT, TST, FST and reversed CUMS-reduced sucrose preference in SPT. In addition, echinacoside reduced the levels of pro-inflammatory cytokines and Iba 1. Moreover, echinacoside significantly increased p-CREB/CREB ratio and BDNF level in hippocampus. Furthermore, echinacoside reduced the secretion of inflammatory factors and inhibited microglia M1 polarization in N9 cells. In conclusion, echinacoside may be beneficial for the treatment of depression diseases through regulating the microglia balance by inhibiting the polarization of microglia to M1 phenotype, and improving hippocampal neurogenesis by the CREB-BDNF signaling pathway.

## 1 Introduction

Depression is a highly prevalent chronic mental disorder that impairs mood and affects people of different ages, races, ethnicities, and genders, resulting in adverse effects on physical health (Saba, Moussavi et al., 2007). At present, a number of factors, such as neurotransmitter deficiency, genetic, environmental, and abnormal neuroimmune processes, endocrine factors, impaired neuronal plasticity and neurogenesis, have been identified as mechanisms to explain the etiology and pathophysiology of depression (Jesulola et al., 2018). There is increasing evidence to support that neuro-inflammatory abnormality may contribute to the pathophysiology of depression (Janssen et al., 2010). Clinically depressed patients manifest higher levels of inflammatory biomarkers, including interleukin (IL)-1β, tumor necrosis factor-alpha (TNF-α), and IL-6 (Del Grande da Silva et al., 2016). In addition, it has been shown that mice or rats with depression induced by lipopolysaccharide (LPS) or exposure to chronic unpredictable mild stress exhibit elevated proinflammatory cytokines (Ge et al., 2015; Xie et al., 2020). Therefore, the neuro-inflammatory disturbance has been acknowledged as a potential avenue of depression. Neuro-inflammatory processes are characterized by the activation of central nervous system (CNS) resident immune effector cells, including microglia. Microglia play a key role in maintaining the microenvironment by recognizing and removing external pathogens *via* phagocytosis and repairing tissues by producing cytokines and chemokines that promote the development of the nervous system (Frost and Schafer, 2016; Weinhard et al., 2018). Previous studies have proven that activated microglia-induced neuroinflammation is linked to the physiopathology of major depressive disorders (Dheen et al., 2007; Brites and Fernandes, 2015).

*Rehmannia glutinosa* Libosch is a well-known medicinal plant in China. It was originally recorded in Shennong Materia Medica (102–200AD, reprinted in 1955), and was listed as the top grade. In the famous traditional Chinese prescription book of Jin Kui Yao Lue, *Rehmannia glutinosa* Libosch was recorded as a kind of antidepressant medicine for the first time. Some antidepressant formulas containing *Rehmannia glutinosa* Libosch, such as Baihe Dihuang Decoction, have been used in China for more than a thousand year. echinacoside (ECH) is a kind of phenylethanoid glycoside isolated from the extract of *Rehmannia glutinosa* Libosch (Li et al., 2020). echinacoside has been extensively studied and was found to have many pharmacological effects such as antioxidant, anti-inflammatory, anti-infective and anti-tumour (Tang et al., 2016). The aim of this research was to study whether echinacoside could ameliorate the depression and to investigate the mechanisms. The CUMS animal model was established to investigate if echinacoside could ameliorate the depression-like behaviors in mice. Then, an *in vitro* model of neuro-inflammation was used to study whether echinacoside could inhibit neuro-inflammation, and to explore whether its neuroprotective effect is related to its anti-inflammatory effect.

## 2 Results

### 2.1 Effects of ECH on CUMS-Induced depressive-like behaviors

**FIGURE 1:**
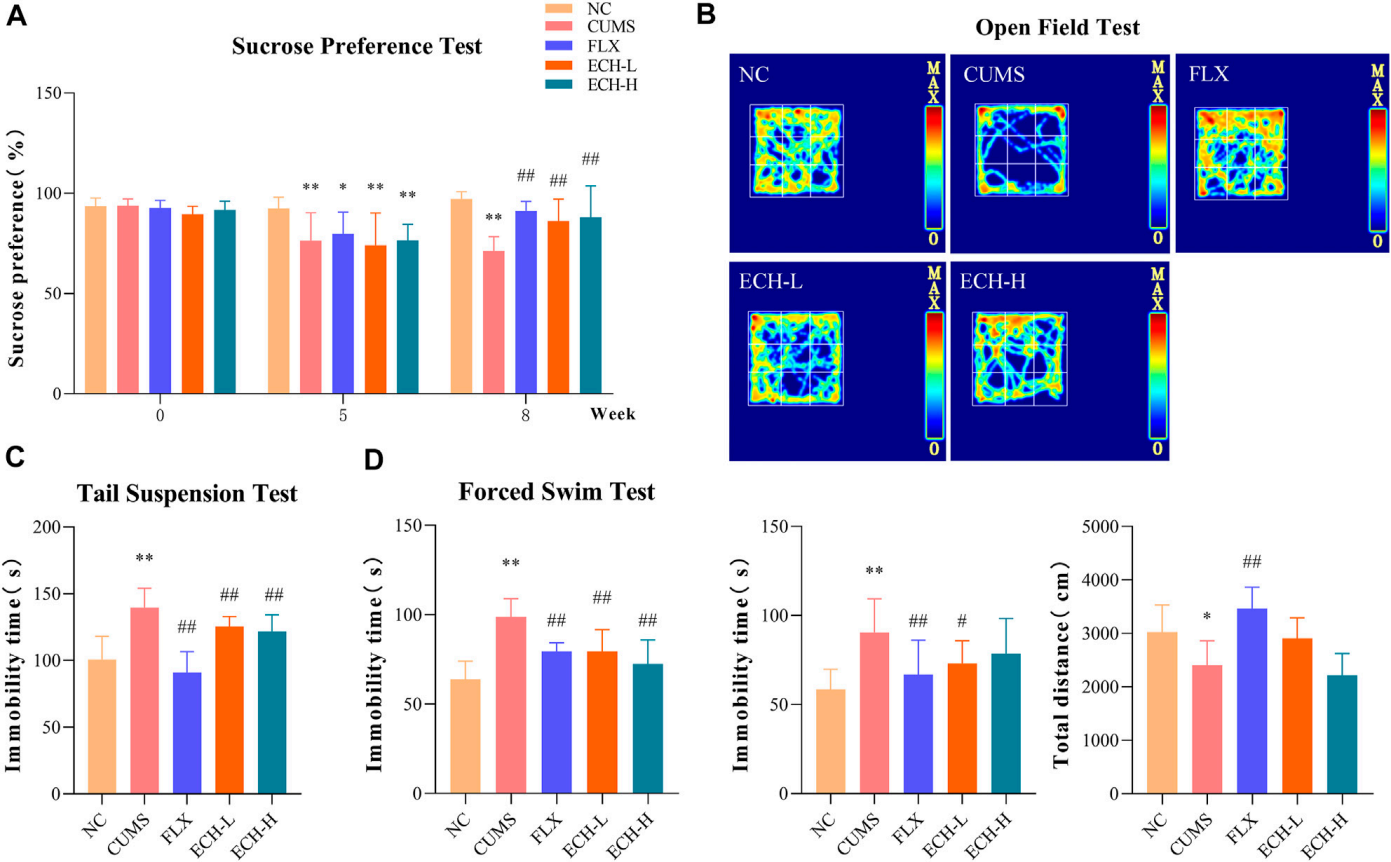
Effects of echinacoside (ECH) on chronic unpredictable mild stress (CUMS)-induced depressive-like behaviors. NC, un-treated normal mice; CUMS, mice were treated with the chronic stimulating factors for 7 weeks to set up the CUMS depression model; FLX, CUMS induced mice were administered with fluoxetine (10 mg/kg,i.g.) daily for 21 days; ECH-L, CUMS induced mice were administered with echinacoside (15 mg/kg,i.g.) daily for 21 days; ECH-H, CUMS induced mice were administered with echinacoside (60 mg/kg,i.g.) daily for 21 days. **(A)** sucrose preference (%) in sucrose preference test (SPT); **(B)** immobility time (s) and total distance in open field test (OFT); **(C)** immobility time (s) in tail suspension test (TST); **(D)** immobility time (s) in forced swimming test (FST). n = 6/group. **p < .01 compared with control group; ^#^ *p* < .05, ^##^ *p* < .01 compared with CUMS group.

As shown in Figure 1A, CUMS-challenged mice exhibited a marked reduction in sucrose preference index compared to normal mice in the sucrose preference test (SPT). After 8 weeks of echinacoside treatment, the sucrose preference index was significantly increased. As shown in Figure 1B, the CUMS mice had a decreased desire for exploration, leading to less total distance travelled and longer immobility time compared to control mice in the open field test (OFT). 15 mg/kg of echinacoside reversed these behavioral alterations, leading to increased total distance travelled and lowered immobility time compared to the CUMS group. As shown in Figure 1C, results showed that CUMS caused a significant increase in immobility duration compared with control mice, while administration of echinacoside (15 mg/kg and 60 mg/kg) significantly reversed the CUMS-induced increase in immobility duration in the TST. Similar findings were obtained in the forced swimming test (FST). CUMS-challenged animals showed a significant increase in immobility duration compared to the control mice, but both 15 mg/kg and 60 mg/kg of echinacoside increased swimming time and reversed the CUMS-induced increase in immobility duration (Figure 1D). These results suggested that echinacoside ameliorated the depression-like behaviors of CUMS mice.

### 2.2 Effects of ECH on hippocampal pathological changes in mice with CUMS-Induced depression

**FIGURE 2:**
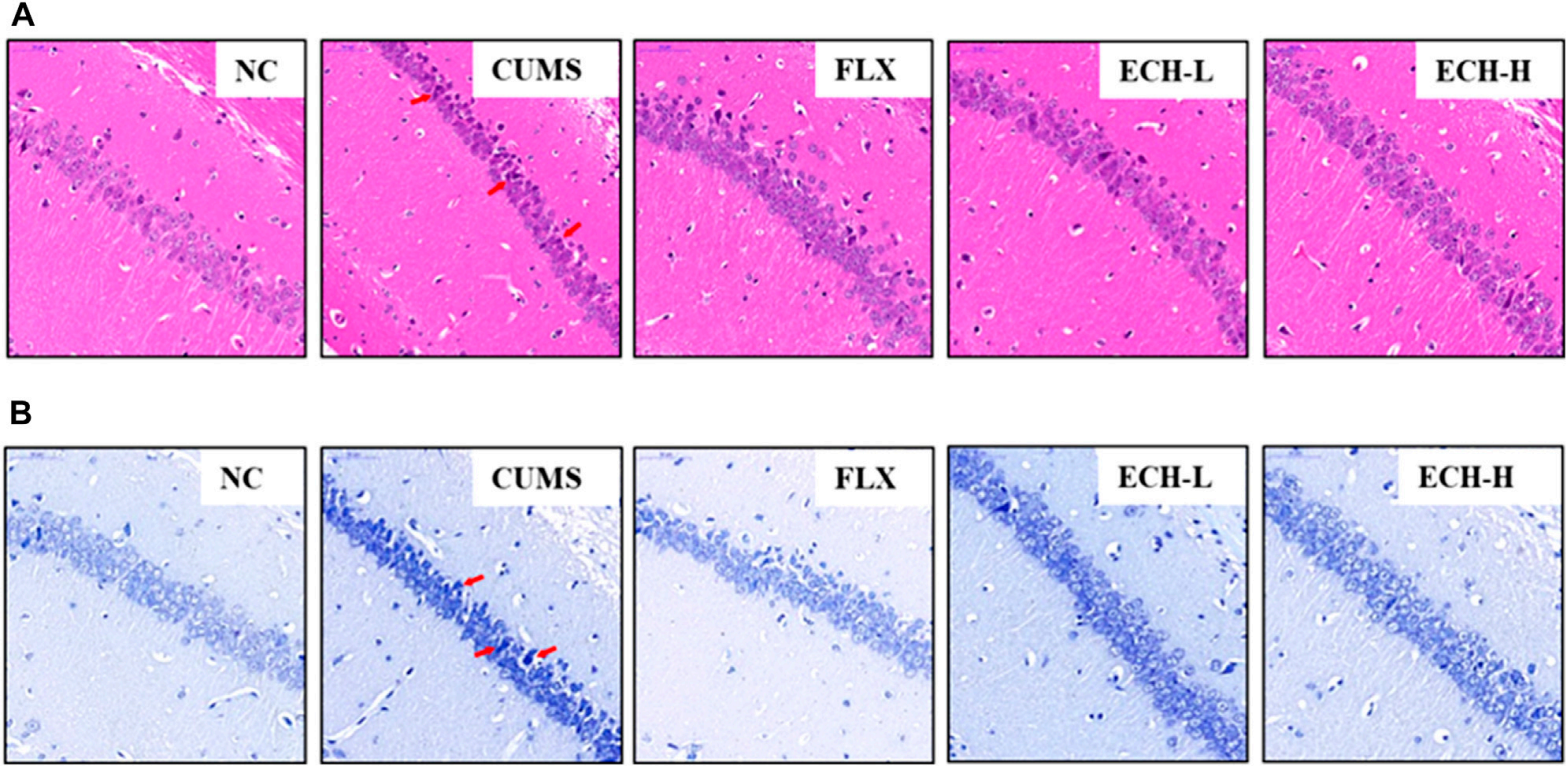
Effects of ECH on hippocampal Pathological Changes in CUMS Mice. NC, un-treated normal mice; CUMS, mice were treated with the chronic stimulating factors for 7 weeks to set up the CUMS depression model; FLX, CUMS induced mice were administered with fluoxetine (10 mg/kg,i.g.) daily for 21 days; ECH-L, CUMS induced mice were administered with echinacoside (15 mg/kg,i.g.) daily for 21 days; ECH-H, CUMS induced mice were administered with echinacoside (60 mg/kg,i.g.) daily for 21 days. **(A)** hematoxylin and eosin (HE) staining of CA1 subfields (400× magnification), scale bar = 50 μm; **(B)** Nissl staining of CA1 subfields (400× magnification), scale bar, 50 μm.

Hematoxylin and eosin (HE) staining (Figure 2A) showed that the hippocampal neurons of normal mice had intact cell structures and were well arranged. The CUMS mice exhibited neuronal pyknosis, partial nuclear fragmentation, and nucleolar blurring and even disappearance. The echinacoside group showed alleviation of the hippocampal neuron damage compared with the model group. Nissl staining showed that the cyton of the neurons was shrunk and neuronal degeneration was observed (Figure 2B), but echinacoside alleviated CUMS-induced neuronal injury.

### 2.3 Effects of ECH on neurotransmitter levels in mice with CUMS-Induced depression

**FIGURE 3:**
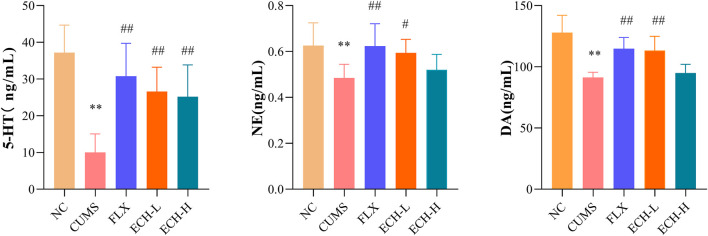
Effects of ECH on 5-HT, NE and DA levels in the hippocampus of CUMS Mice. NC, un-treated normal mice; CUMS, mice were treated with the chronic stimulating factors for 7 weeks to set up the CUMS depression model; FLX, CUMS induced mice were administered with fluoxetine (10 mg/kg,i.g.) daily for 21 days; ECH-L, CUMS induced mice were administered with echinacoside (15 mg/kg,i.g.) daily for 21 days; ECH-H, CUMS induced mice were administered with echinacoside (60 mg/kg,i.g.) daily for 21 days *n* = 6/group. **p < .01 compared with control group; ^#^ *p* < .05, ^##^ *p* < .01 compared with CUMS group.

The levels of 5-hydroxytryptamine (5-HT), norepinephrine (NE), and dopamine (DA) detected in the hippocampus are summarized in Figure 3. Compared with the control group, the CUMS group revealed a significant decrease in 5-HT, NE, and DA levels in the hippocampus, but treatment with 15 mg/kg echinacoside increased the levels of these neurotransmitters.

### 2.4 Effects of ECH on HPA axis function in mice with CUMS-Induced depression

**FIGURE 4:**
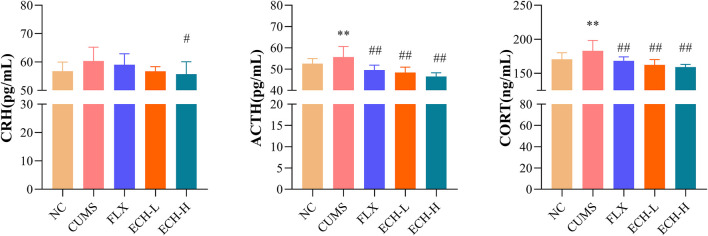
Effects of ECH on CRH, ACTH and CORT levels in the serum of CUMS Mice. NC, un-treated normal mice; CUMS, mice were treated with the chronic stimulating factors for 7 weeks to set up the CUMS depression model; FLX, CUMS induced mice were administered with fluoxetine (10 mg/kg,i.g.) daily for 21 days; ECH-L, CUMS induced mice were administered with echinacoside (15 mg/kg,i.g.) daily for 21 days; ECH-H, CUMS induced mice were administered with echinacoside (60 mg/kg,i.g.) daily for 21 days *n* = 8/group. ^*^ *p* < .05, **p < .01 compared with control group; ^#^ *p* < .05, ^##^ *p* < .01 compared with CUMS group.

As shown in Figure 4, the HPA axis abnormalities were observed in the CUMS group. The serum corticotropin-releasing hormone (CRH), adrenocorticotrophic hormone (ACTH), and corticosterone (CORT) levels of the CUMS group were evidently higher than those of the control group. Compared with the CUMS group, CRH, ACTH, and CORT levels of the echinacoside groups (15 mg/kg and 60 mg/kg) were significantly decreased.

### 2.5 Effects of ECH on inflammatory factors in the hippocampus in mice with CUMS-Induced depression

**FIGURE 5:**
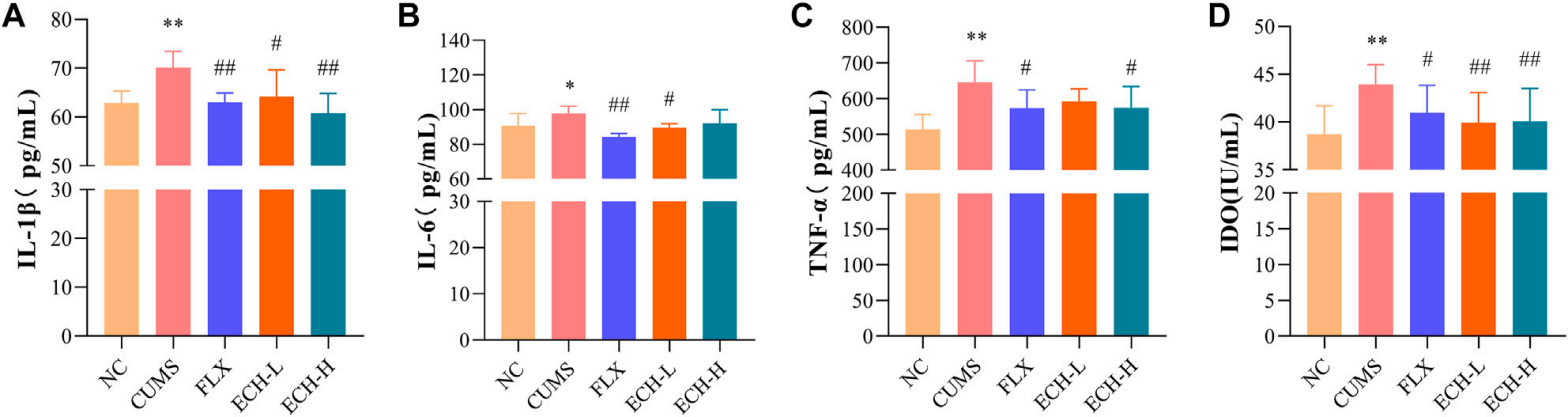
Effects of ECH on IL-1β **(A)**, IL-6 **(B)**, TNF-α **(C)** and IDO **(D)** levels in the hippocampus of CUMS Mice. NC, un-treated normal mice; CUMS, mice were treated with the chronic stimulating factors for 7 weeks to set up the CUMS depression model; FLX, CUMS induced mice were administered with fluoxetine (10 mg/kg,i.g.) daily for 21 days; ECH-L, CUMS induced mice were administered with echinacoside (15 mg/kg,i.g.) daily for 21 days; ECH-H, CUMS induced mice were administered with echinacoside (60 mg/kg,i.g.) daily for 21 days. *n* = 6/group. *p < .05, **p < .01 compared with control group; ^#^ *p* < .05, ^##^ *p* < .01 compared with CUMS group.

IL-1β, IL-6, and TNF-α levels in the hippocampus were measured by ELISA. The CUMS treatment caused the increases in IL-1β, IL-6, and TNF-α, while 15 mg/kg and 60 mg/kg of echinacoside significantly decreased the IL-1β and IL-6 levels, and 15 mg/kg of echinacoside significantly decreased the TNF-α level in the hippocampus (Figures 5A, B, C). Indoleamine 2,3-dioxygenase (IDO) is an enzyme activated by pro-inflammatory cytokines. IDO activity of the CUMS group was increased compared to the control group. Compared with the CUMS group, treatment with 15 mg/kg and 60 mg/kg of echinacoside decreased IDO level significantly (Figure 5D).

### 2.6 Effects of ECH on Iba-1 and iNOS expression levels in mice with CUMS-Induced depression

**FIGURE 6:**
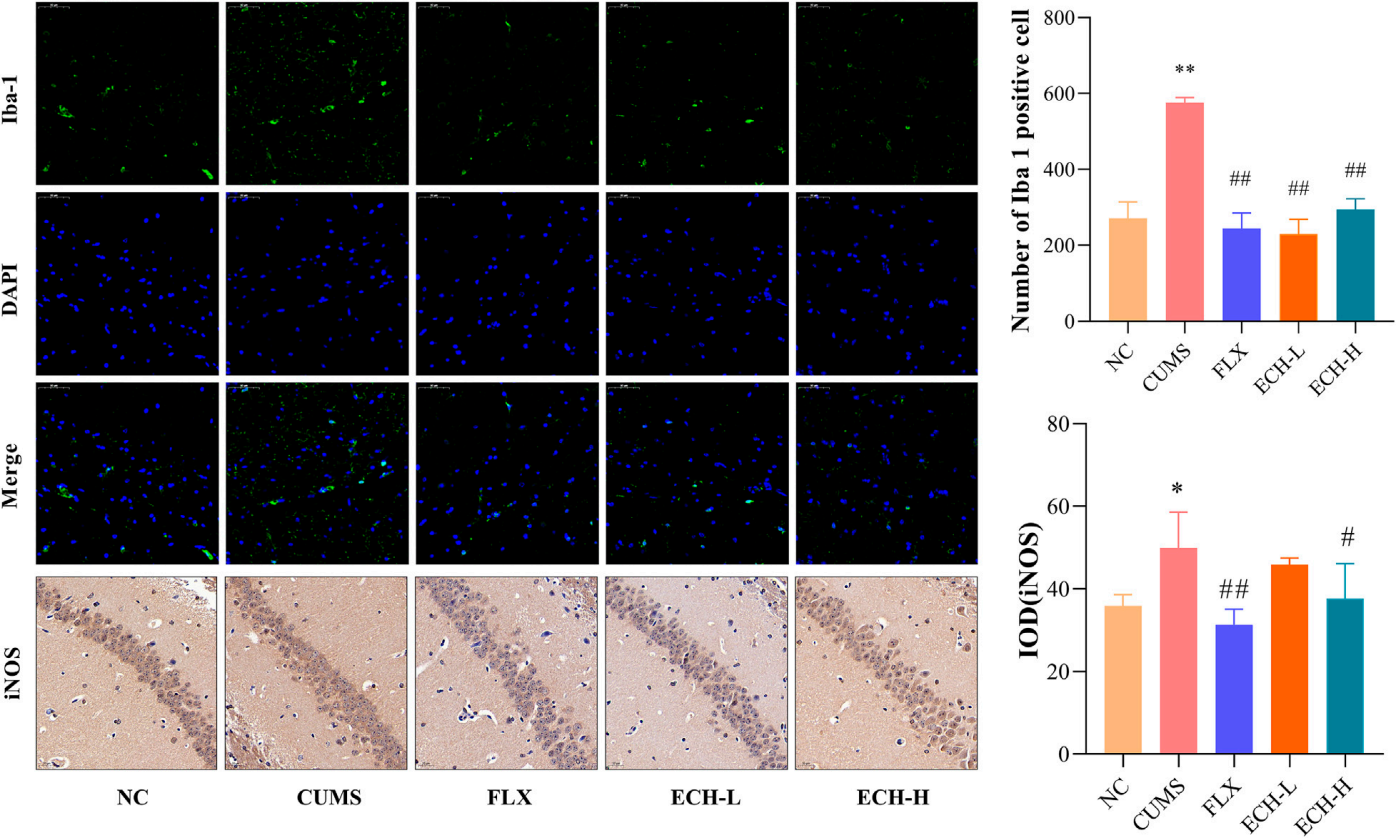
Effect of ECH on Iba-1 and iNOS protein expression in the hippocampus of CUMS Mice. NC, un-treated normal mice; CUMS, mice were treated with the chronic stimulating factors for 7 weeks to set up the CUMS depression model; FLX, CUMS induced mice were administered with fluoxetine (10 mg/kg,i.g.) daily for 21 days; ECH-L, CUMS induced mice were administered with echinacoside (15 mg/kg,i.g.) daily for 21 days; ECH-H, CUMS induced mice were administered with echinacoside (60 mg/kg,i.g.) daily for 21 days *n* = 3/group. *p < .05, **p < .01 compared with control group; ^#^ *p* < .05, ^##^ *p* < .01 compared with CUMS group.

Microglial cells play an essential role in neuro-inflammatory processes in the brain. Iba-1 is widely used as a microglia activation marker (Liu et al., 2020). When inflammation occurs, pro-inflammatory mediators such as NO, which is synthesi sed by iNOS, are released. Results showed that the CUMS procedure increased the amount of iNOS and the number of Iba1^+^ microglia in hippocampus compared with the control group. High doses of echinacoside significantly reduced iNOS content and both high and low doses of echinacoside significantly reduced Iba1+ microglia compared to mice with CUMS-induced depression-like behaviour (Figure 6).

### 2.7 Effects of ECH on JAK1/STAT3 pathway in the hippocampus in mice with CUMS-Induced depression

**FIGURE 7:**
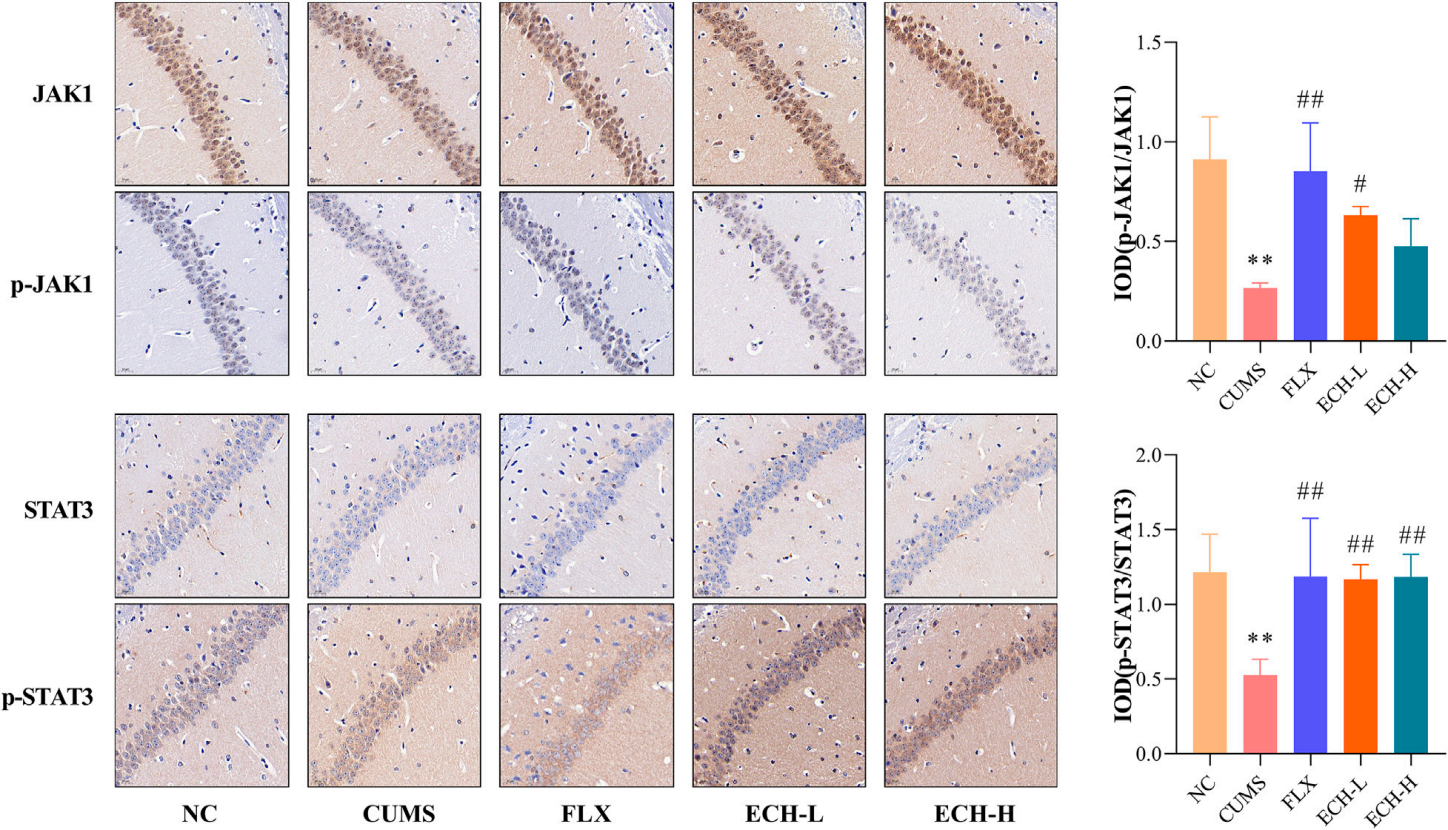
Effects of ECH on p-JAK1/JAK1 and p-STAT3/STAT3 levels in the hippocampus of CUMS Mice. NC, un-treated normal mice; CUMS, mice were treated with the chronic stimulating factors for 7 weeks to set up the CUMS depression model; FLX, CUMS induced mice were administered with fluoxetine (10 mg/kg,i.g.) daily for 21 days; ECH-L, CUMS induced mice were administered with echinacoside (15 mg/kg,i.g.) daily for 21 days; ECH-H, CUMS induced mice were administered with echinacoside (60 mg/kg,i.g.) daily for 21 days *n* = 3/group. **p < .01 compared with control group; ^#^ *p* < .05, ^##^ *p* < .01 compared with CUMS group.

The JAK1/STAT3 signaling pathway is a common pathway involving in the proliferation, differentiation, apoptosis and inflammatory response of neuronal cells. Results showed that the p-JAK1/JAK1raito and p-STAT3/STAT3 ratio were significantly decreased in the hippocampus of mice in the CUMS group compared to the NC group. Compared with the CUMS group, 15 mg/kg of echinacoside increased p-JAK1/JAK1 ratio significantly, 15 mg/kg and 60 mg/kg of echinacoside increased p-STAT3/STAT3 ratio significantly (Figure 7).

### 2.8 Effects of ECH on CREB–BDNF signal pathway in mice with CUMS-Induced depression

**FIGURE 8:**
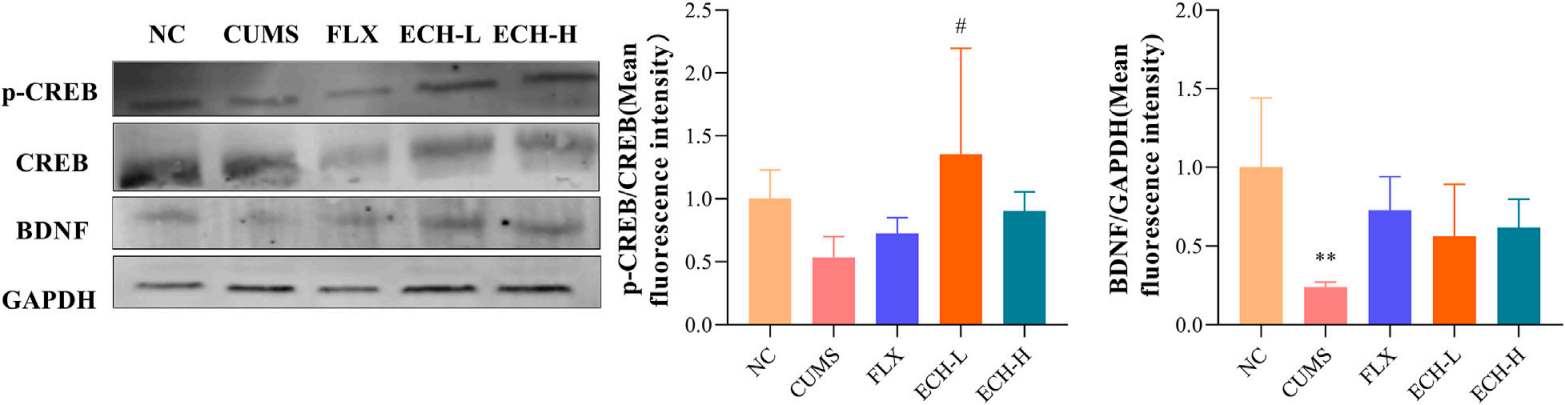
Effects of ECH on key protein expressions in the CREB/BDNF pathway in hippocampus of CUMS Mice. NC, un-treated normal mice; CUMS, mice were treated with the chronic stimulating factors for 7 weeks to set up the CUMS depression model; FLX, CUMS induced mice were administered with fluoxetine (10 mg/kg,i.g.) daily for 21 days; ECH-L, CUMS induced mice were administered with echinacoside (15 mg/kg,i.g.) daily for 21 days; ECH-H, CUMS induced mice were administered with echinacoside (60 mg/kg,i.g.) daily for 21 days *n* = 3/group. **p < .01 compared with control group; ^#^ *p* < .05 compared with CUMS group.

After treated with CUMS, p-cAMP responsive element binding protein/cAMP-response element binding protein (p-CREB/CREB) and brain-derived neurotrophic factor (BDNF) were markedly decreased in the hippocampus, whereas treatment with echinacoside resulted in significant increases of p-CREB/CREB ratio and BDNF level in hippocampus compared with CUMS group (Figure 8).

### 2.9 Effects of ECH on cell viability and NO production in N9 microglial cells induced by LPS

**FIGURE 9:**
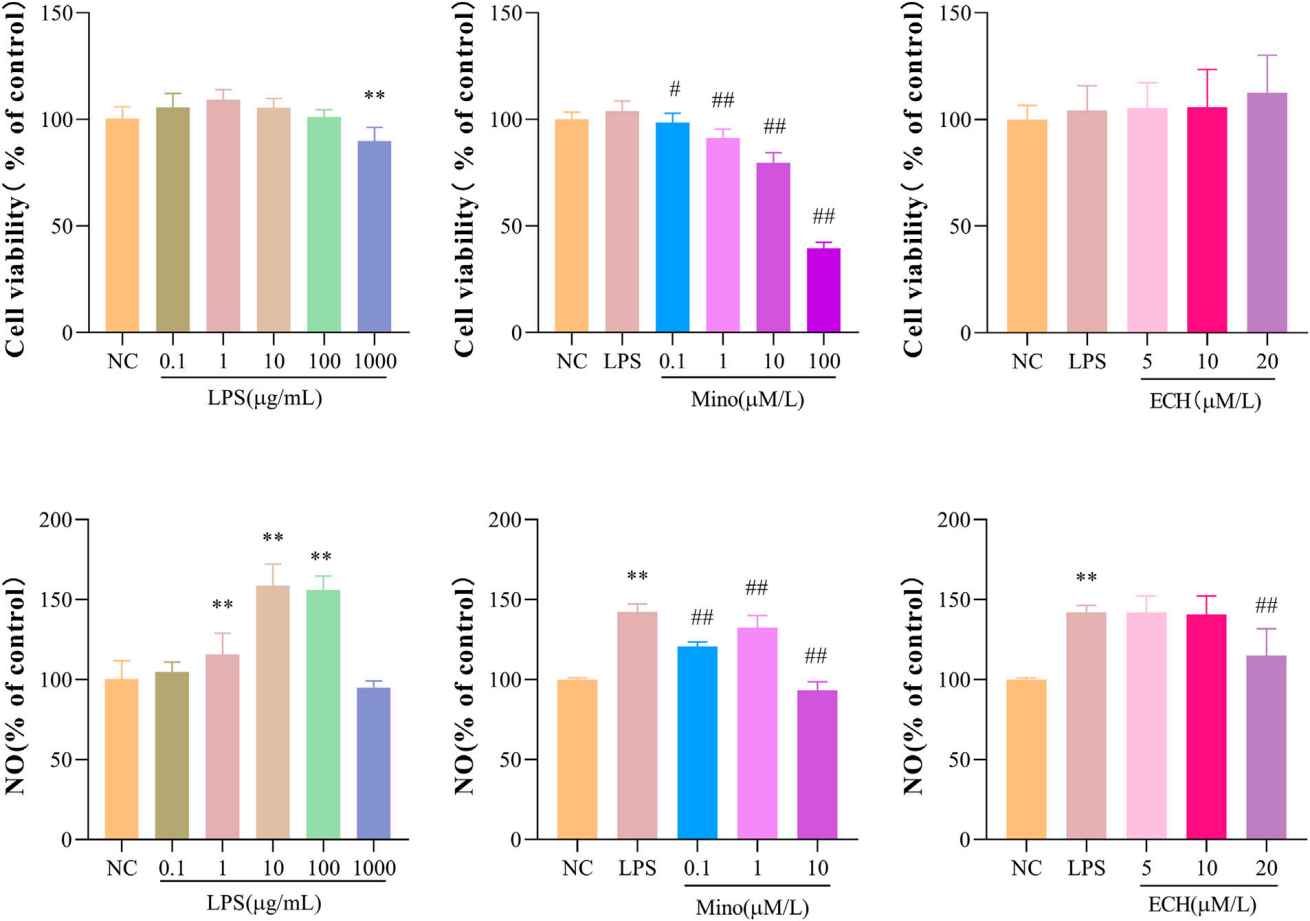
Effects of ECH on Cell Viability and NO Production in LPS induced N9 cells. **(A)** Cell viability of N9 microglial cells incubated with .1, 1, 10, 100, and 1000 μg/ml of lipopolysaccharide (LPS) for 24 h; **(B)** Cell viability of N9 microglial cells stimulated with .1, 1, 10 μM of minocycline (Mino) and 1 μg/ml LPS for 24 h; **(C)** Cell viability of N9 microglial cells treated with 5, 10, and 20 μM of ECH and 1 μg/ml LPS for 24 h. **(D)** NO production of N9 microglial cells incubated with .1, 1, 10, 100, and 1000 μg/ml of LPS for 24 h; **(E)** NO production of N9 microglial cells stimulated with .1, 1, 10 μM of Mino and 1 μg/ml LPS for 24 h; **(F)**NO production of N9 microglial cells treated with 5, 10, and 20 μM of ECH and 1 μg/ml LPS for 24 h. Results are expressed as percentage values, taking control group as 100%. *n* = 6/group. **p < .01 compared with control group; ^#^ *p* < .05, ^##^ *p* < .01 compared with LPS group.

Methylthiazol tetrazolium (MTT) assay was used to evaluate whether the administered dose of echinacoside and LPS had effects on N9 cell viability. The results showed that incubation with LPS at concentrations of .1, 1, 10, and 100 μg/mL alone for 24 h had no significant effect on the cell viability of the N9 microglia, but 1000 μg/mL LPS significantly reduced the cell viability compared to the control group (Figure 9A). In addition, the combined treatment of minocycline (Mino, .1 μM) and LPS (1 μg/mL) or the combined treatment of echinacoside (5, 10, and 20 μM) and LPS (1 μg/mL) for 24 h did not affect the cell viability of N9 microglia compared with the control group (Figures 9B, C). Griess’ method was used to measure the NO level. Compared with the control group, stimulation of N9 cells with LPS (1, 10, 100 μg/mL) resulted in a marked increase in NO production (Figure 9D). In addition, minocycline (.1, 1, 10 μm) significantly reduced NO production compared to LPS group (Figure 9E). echinacoside at concentrations of 5 and 10 μm had no significant effects on NO production, while 20 μm echinacoside significantly reduced NO production (Figure 9F). Therefore, N9 microglial cells were exposed to 1 μg/ml LPS to induce microglial activation and .1 μm Mino was used as the positive control. 20 μm echinacoside might suppress the inflammation without decrease cell viability in N9 microglia induced by LPS.

### 2.10 Effect of ECH on morphology and iba one expression in N9 microglial cells induced by LPS

**FIGURE 10:**
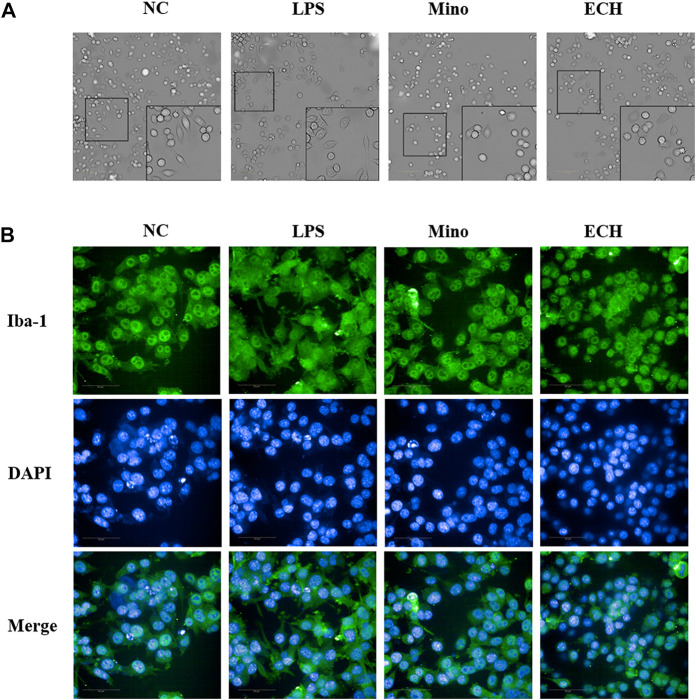
Effects of ECH on morphology **(A)** and Iba 1 expression level **(B)** in LPS-induced N9 cells. NC, treated with equal amounts of medium; LPS, treated with1 μg/mL LPS; Mino, treated with 1 μg/mL LPS and .1 μM minocycline; ECH, treated with 1 μg/ml LPS and 20 μM echinacoside. n = 3/group. ^*^ *p* < .05, **p < .01 compared with control group.

In the control group, microglia are in a resting state. After treated by LPS for 24 h, activated microglial cells with the characteristics of enlarged cell body and synaptic retraction were observed. echinacoside inhibited LPS-induced morphology changes of N9 microglia, as observed by smaller soma and slender process compared to the LPS-treated group (Figure 10A). The Iba 1 level of N9 microglia was analyzed by high-content screening assay to further explore the status of activation of microglia. Results showed that the level of Iba 1 in the LPS group increased significantly compared to the control group and echinacoside suppressed Iba 1 expression significantly compared with the LPS group (Figure 10). Results suggested that echinacoside attenuated the activation of microglia.

### 2.11 Effects of ECH on secretion of inflammatory factors and anti-inflammatory factors in N9 microglial cells induced by LPS

**FIGURE 11:**
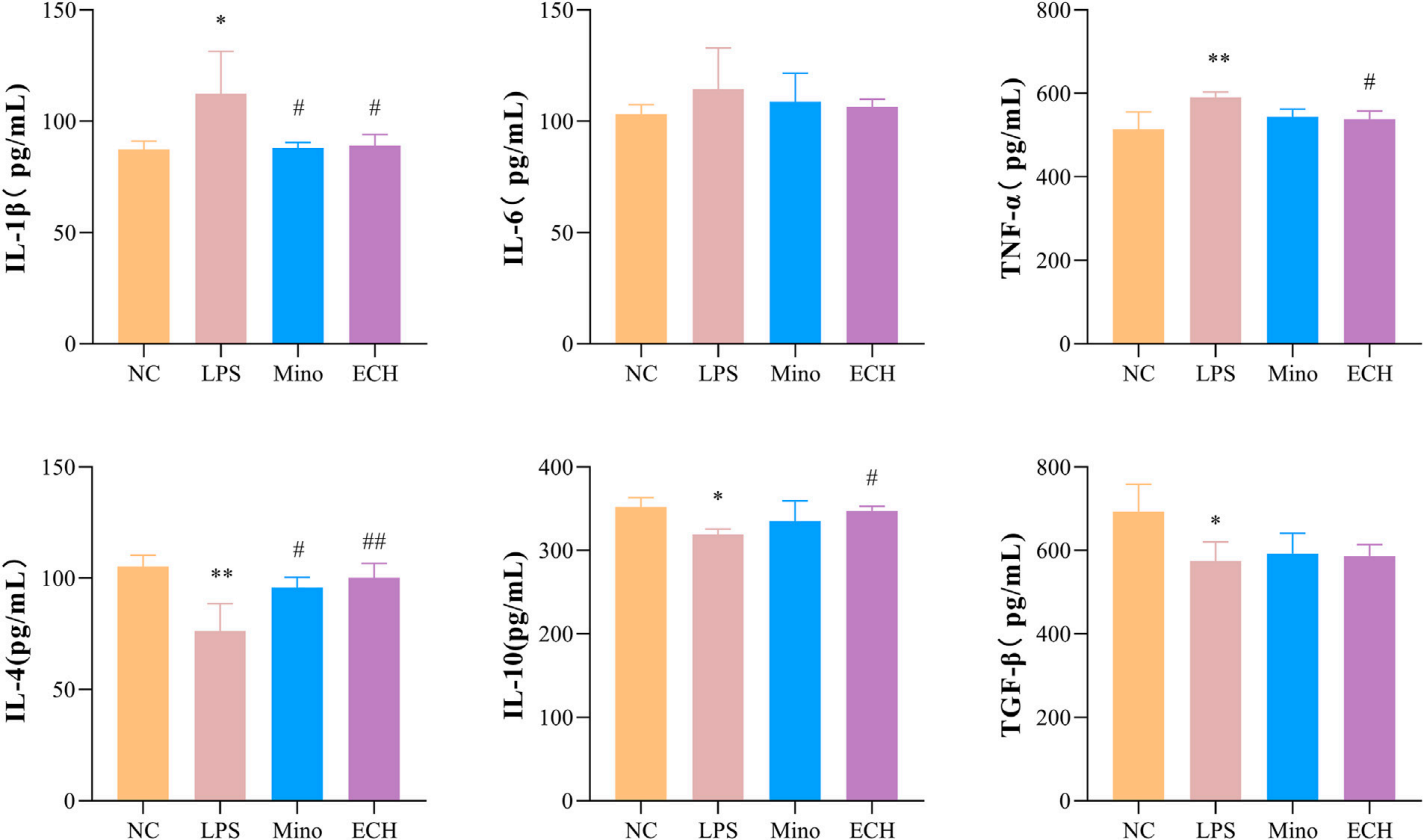
Effects of ECH on IL-1β, IL-6, TNF-α, IL-4, IL-10, and TGF β levels in LPS induced N9 cells. NC, treated with equal amounts of medium; LPS, treated with 1 μg/ml LPS; Mino, treated with 1 μg/ml LPS and .1 μM minocycline; ECH, treated with 1 μg/ml LPS and 20 μM echinacoside. n = 3/group. *p < .05, **p < .01 compared with control group; ^#^ *p* < .05, ^##^ *p* < .01 compared with LPS group.

To investigate whether echinacoside can antagonize LPS-induced inflammatory responses, inflammatory factors and anti-inflammatory factors in the supernatant of N9 cells were quantified by enzyme-linked immunosorbent assay (ELISA). Pro-inflammatory factors (IL-1β, TNF-α) in cells treated with LPS were significantly up-regulated compared with the control group. 20 μM of echinacoside effectively reversed the increase IL-1β and TNF-α productions induced by LPS (Figure 11). Furthermore, anti-inflammatory factors (IL-4 and TGF β) in the LPS group were significantly decreased compared with the control group, and echinacoside up-regulated the anti-inflammatory factors of IL-4 and IL-10 slightly (no significant difference) compared with the LPS group in N9 microglia (Figure 11). These results revealed that echinacoside regulated the polarization of microglia from M1 to M2 phenotype.

### 2.12 Effects of ECH on M1 and M2 Microglial Markers in N9 microglial cells induced by LPS

**FIGURE 12:**
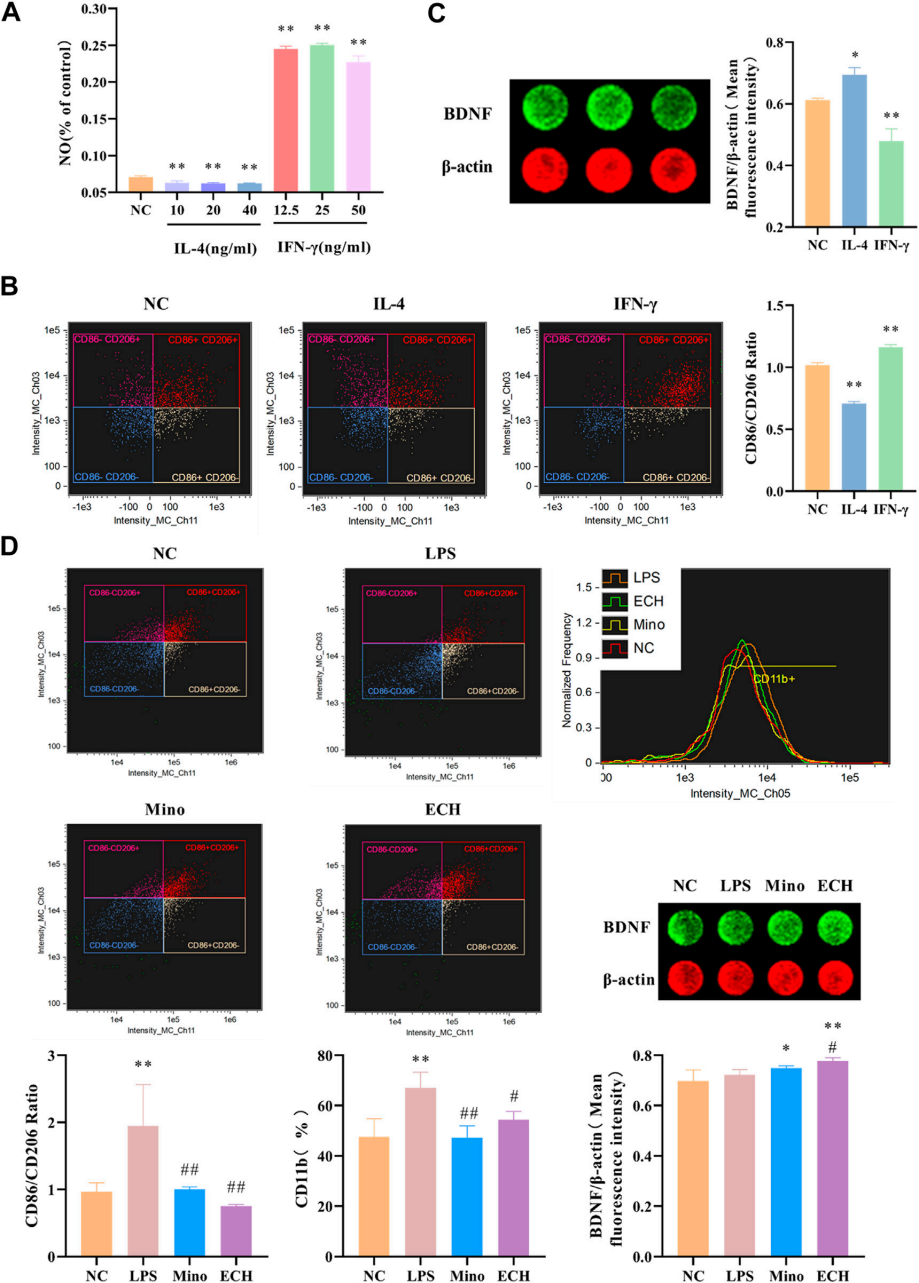
Effects of ECH on M1 and M2 Microglial Markers and BDNF level in LPS induced N9 cells. **(A)** Effect of IL-4 and IFN-γ on NO level in the supernatant of N9 cells. **(B)** Effects of IL-4 and IFN-γ on CD11b, CD86, and CD206 in N9 cells. **(C)** Effects of IL-4 and IFN-γ on BDNF level in N9 cells. **(D)** Effects of ECH on CD11b, CD86, CD206 and BDNF levels in LPS induced N9 cells. NC, treated with equal amounts of medium; IL-4, treated with 40 ng/ml IL-4; IFN-γ, treated with 25 ng/ml IFN-γ; LPS, treated with 1 μg/ml LPS; Mino, treated with 1 μg/ml LPS and .1 μM minocycline; ECH,treated with 1 μg/ml LPS and 20 μM echinacoside. n = 4/group **(A)**, n = 3/group **(B,C,D)**. ^*^ *p* < .05, **p < .01 compared with control group; ^#^ *p* < .05, ^##^ *p* < .01 compared with LPS group.

Results showed that IL-4, a Th1-type cytokine, was able to significantly reduce NO levels in cell supernatants, while IFN-γ, a Th1-type cytokine, was able to significantly increase NO levels in cell supernatants (Figure 12A). The levels of M1 marker (CD11b, CD86) and M2 marker (CD206) were detected by imaging flow cytometry, and results showed that IL-4 was able to induce the polarization of microglia toward the M2 phenotype, while IFN-γ induce the polarization of microglia toward the M2 phenotype. (Figure 12B). Moreover, results showed that the BDNF expression level in N9 microglia cells polarized into M2 phenotype was higher than that in N9 microglia cells polarized into M1 phenotype (Figure 12C). Meanwhile, the results showed that LPS induce the polarization of microglia toward the M1 phenotype, which is similar to IFN-γ. echinacoside inhibited the CD11b expression level on N9 microglia compared with the LPS-treated group, and down-regulated the CD86/CD206 ratio significantly compared with the LPS-treated group (Figure 12D), revealing that echinacoside could regulate the polarization of microglia from M1 to M2. At the same time, the results showed that echinacoside could significantly elevate intracellular BDNF levels (Figure 12).

## 3 Discussion

Recent accumulating evidence suggested that echinacoside has neuroprotective effects and is widely studied in Alzheimer’s disease, osteoporosis (Keller et al., 2017; Chen et al., 2020), Parkinson’s disease (Liang et al., 2019), hypoxic pulmonary hypertension (Gai et al., 2020), cognitive impairment (Ding et al., 2022), hypoxic-ischemic brain injury (Wei et al., 2019), diabetes (Kong et al., 2018), inflammation (Tan and Zhang, 2022), *et al.* Depression is associated with decreased neurologic function and new brain lesions. In addition, more and more studies in recent years have mentioned that the pathogenesis of depression is closely linked to neuro-inflammation. Therefore, the aim of this research was to study whether echinacoside could ameliorate the depression and to investigate its mechanisms involving the neuro-inflammation and neurotrophin signaling pathway. In this study, echinacoside increased sucrose preference during OFT, and decreased immobility time of mice in TST, and FST in the CUMS-induced mice. Chronic stress has been shown to negatively regulate adult hippocampal neurogenesis (Mahar et al., 2014). Results showed echinacoside alleviated the hippocampal neuron damage in the CUMS-induced mice. These results revealed that echinacoside could ameliorate the depression.

Stress response-induced the hypothalamic-pituitary-adrenal (HPA) axis hyperactivity and neurotransmitter deficiency are two major mechanisms related to the onset and development of depression (Peng et al., 2015). The imbalance between central corticosteroid receptors is proposed to underlie dysregulation of the HPA axis and vulnerability to stress-related psychiatric disorders, like anxiety, depression, posttraumatic stress disorder. The feedback mechanism of the HPA axis is closely linked to glucocorticoids in the brain and pituitary gland. Glucocorticoids act through two receptors: the high affinity mineralocorticoid receptor (MR) and the lower affinity glucocorticoid receptor (GR). MR and GR are abundant in the limbic system (e.g. hippocampus), and mediate the initiation and termination of the HPA axis stress response *via* negative feedback, and modulate acquisition, processing, storage and retrieval of stressful experiences. Imbalance between MR and GR-mediated actions in limbic system neurons may lead to an exaggerated or inadequate initial HPA axis response to stress, finally increase vulnerability to affective disease (Harris et al., 2013). Growing evidence has shown decreased levels of monoamine transmitters (NE, 5-HT, and DA) and HPA axis function (ACTH、CORT、CRH) disorder in the brains of depressed animals (Wang et al., 2019). Therefore, cortisol level, HPA axis function and neurotransmitter were studied in this research. In this research, echinacoside reversed the elevated CORT, ACTH, and CRH levels in CUMS mice, and 15 mg/kg echinacoside induced a significant increase in 5-HT, NE, and DA levels. These results suggested that echinacoside may have antidepressant-like action.

Inflammation is the first response of the immune system to infection or stimulation and plays an important role in the development and progression of many diseases. It can be broadly defined as the recruitment of lymphoid and myeloid cells to a site of injury or infection, and cytokines are integral to the development of inflammation. CUMS-induced depression is accompanied by neuro-inflammatory response. Peripheral cytokines can be involved in the pathways that affect the central synthesis of HPA hormones and neurotransmitters (Serna-Rodríguez et al., 2022). TNF-α might contribute to the pathogenesis of depression by activation of the HPA axis. On the other hand, during an acute depressive episode, elevated HPA axis activity may suppress TNF-α system activity (Berthold-Losleben and Himmerich, 2008). In the present study, the antidepressant-like action of echinacoside might be the result of the interaction between suppression of inflammatory factors and activation of HPA axis. When inflammation occurs, macrophages are activated and induced to produce pro-inflammatory mediators such as nitric oxide (NO), which is produced by inducible nitric oxide synthase (iNOS) (Xiong et al., 2014). Inhibition of the production of pro-inflammatory mediators is an important way to combat inflammation. Our data showed that the increased iNOS in the hippocampus of CUMS mice could be inhibited by echinacoside. Microglia are important innate macrophage-like immune cells in the central nervous system. During homeostasis, microglia are in a “resting” state, maintaining the stability of the central nervous system by monitoring pathological changes in the brain parenchyma and engulfing cellular debris such as plaques and damaged or unwanted synapses (Chung and Barres, 2012). It will be activated when the brain suffers from inflammation, infection, or other neurological insults, and play an important role in regulating immune response and inflammation (Kreutzberg, 1996; Jia et al., 2020). It has been reported that the M1 phenotype of microglia leads to HPA axis hyperactivity and neurotransmitter dysfunction (Liu et al., 2021). Pro-inflammatory cytokines released from microglia disrupt the HPA axis and influence the release of 5-hydroxytryptaminergic, noradrenergic, dopaminergic, and glutamatergic neurotransmission. Microglia can express various pro-inflammatory factors (TNF-α, IL-1β and IL-6) after activated to induce neuroinflammation or even cause neuronal death (Motaghinejad et al., 2017). These pro-inflammatory cytokines could increase the activity of IDO (Xu et al., 2015), which is the enzyme that catalyzes the rate-limiting step in the catabolism of tryptophan in the kynurenine (KYN) pathway. 5-hydroxytryptamine (5-HT) is biochemically derived from tryptophan (Lu et al., 2020). Therefore, pro-inflammatory cytokines, including IL-1, IL-6, and TNF-α, may cause 5-HT synthesis deficiency by activation of the tryptophan metabolizing enzyme IDO, and finally lead to depression (Ying et al., 2017). Previous clinical studies pointed out that increased serum and/or plasma concentrations of IL-1β, IL-6, and TNF-α are the most frequently observed inflammatory markers in depressed patients, and there is a positive correlation between IL-1β, IL-6, TNF-α levels and depressive symptoms (Lotrich et al., 2011; Hodes et al., 2015). Increasing evidence indicates that CUMS administration was reported to induce the up-regulation of pro-inflammatory cytokines in the central nervous system (Fu et al., 2019). The present *in vivo* study showed that these inflammatory mediators were up-regulated in CUMS group and down-regulated by echinacoside, which indicated that the increased 5-HT level after echinacoside administration was partly caused by the suppressed IL-1β and IL-6 and inhibited IDO activity by echinacoside. Ionized calcium-binding adapter molecule 1 (Iba1) was used as a marker of microglia activation to judge microglia in the state of activation. Our data are consistent with the previous findings that the number Ye et al., 2020) the Iba1^+^ microglia was increased in the hippocampus of the mice with CUMS-induced depression, and echinacoside decreased the numbers of Iba1^+^ microglia.

Recent studies have revealed that microglia activation is associated with a variety of neuropsychiatric diseases, such as depression (Deng et al., 2020). The LPS-induced N9 cell activation model is a common *in vitro* model for study neuro-inflammation. This experiment was performed according to Ye’s assay to investigate the effects of echinacoside on neuro-inflammation (Ye et al., 2020). A large number of studies pointed out that microglia will be activated accompanied by changes in cell morphology and function if microglia are exposed to a pro-inflammatory (Berthold-Losleben and Himmerich, 2008; Menassa and Gomez-Nicola, 2018). Results showed that N9 microglia activated by 1 μg/ml LPS tended to become amoebic from branching with up-regulated Iba-1 and NO levels. echinacoside administration normalized cell morphology of the N9 microglia and suppressed Iba-1 and NO levels. These findings indicated that echinacoside exerts an anti-depressive effect by inhibiting microglial activation. Microglia may exert both detrimental and beneficial effects on neurogenesis, depending on the morphological and molecular phenotype. Microglia polarization refers to different functional phenotypes after microglia are activated. The classic activated microglia (M1 phenotype) could display pro-inflammatory phenotype, and have negative effects on neurogenesis. In contrast, the alternative activated microglia (M2 phenotype) may show increased expression of anti-inflammatory cytokines and nerve growth factors, and have positive effects on neurogenesis and regenerative processes (Ye et al., 2020). Microglia can be polarized into the classically activated pro-inflammatory M1 phenotype induced by LPS or INF-γ, and can be polarized into the alternatively activated anti-inflammatory M2 phenotype induced by IL-4. The M1 microglia will secrete pro-inflammatory factors (iNOS, IL-1β, IL-6, and TNF-α *et al*), which participates in the process of central nervous system injury. The M2 microglia will secrete anti-inflammatory factors (IL-4, IL-10, and TGF-β *et al*), glial cell derived neurotrophic factors or nerve growth factors, which mediates the process of nerve repair and regeneration of the brain (Zhou et al., 2019). According to the previews researchers, CUMS may contribute to excessive inflammation and skew microglial polarization towards the M1 phenotype, and a high M1/M2 ratio is associated with depression in rats (Guo et al., 2020). Thus, microglia activation and M1/M2 phenotype switching may be involved in the pathological process of depression. In the current experiment, the pro-inflammatory cytokines of IL-1β and TNF-α were significantly higher, and anti-inflammatory cytokines of IL-4 and TGF *β* were lower in LPS group compared to the control group. Meanwhile, CD11b and CD86/CD206 were significantly higher in LPS group compared to the control group. echinacoside administration suppressed LPS-induced elevated levels of IL-1β, TNF-α, and CD11b, CD86/CD206 in N9 microglial cells. The above results suggested that echinacoside may prevent LPS-induced N9 microglia cell polarization to the M1 phenotype by affecting the secretion of inflammatory cytokines.

As cytoplasmic transcription factors, signal transducers and activators of transcription (STAT) proteins mediate extracellular signaling to the nucleus controlling fundamental functions, such as cell proliferation, apoptosis, differentiation, immune responses and angiogenesis. Su *et al* reported that JAK1/STAT3 signaling pathway is important for the proliferation, differentiation, apoptosis and inflammatory response of neuronal cells (Su et al., 2018). Bai’s study showed that the levels of p-JAK1/JAK1 and p-STAT3/STAT3 were significantly decreased in the hippocampal tissue of the depression rats (Bai et al., 2022). Zhang *et al* (Qian and Yang, 2019) reported the JAK1/STAT3 signaling pathway is activated to induce cell proliferation, differentiation and apoptosis in response to brain tissue damage (Zhang et al., 2018). It was also reported that activation of the JAK1/STAT3 signaling pathway could reduce β-amyloid-induced neuronal damage in the rat hippocampus. The results in the present experiment showed that echinacoside was able to elevate the p-JAK1/JAK1 and p-STAT3/STAT3 levels, suggesting that echinacoside could activate the JAK1/Stat3 signaling pathway, consequently promote the growth of neuronal cells and suppress neuro-inflammation. Rei Nakamura *et al* reported that sustained STAT3 signaling in senescent macrophages channels M2 polarization and promotes neovascularization (Nakamura et al., 2015). In this study, JAK1/Stat3 signaling pathway might be involved in microglia polarization to M2 phenotype by echinacoside. More experiments and evidences are needed to support this hypothesis.

Decreased BDNF is another significant factor in the pathogenesis of depression (Castrén and Rantamäki, 2010). Nagahara (Nagahara and Tuszynski, 2011) highlighted the therapeutic potential of BDNF in a range of CNS disorders, including depression. CREB-BDNF signaling has been implicated in regulating several functions, including cell survival, synaptic structure, and synaptic plasticity. Recently, some animal tests have indicated that the CREB-BDNF signaling pathway in the hippocampus is related to depression and the pathogenesis of cognitive function impairments. In the present study, p-CREB/CREB ratio and BDNF expression level decreased in hippocampus of mice with CUMS-induced depression and echinacoside up-regulated BDNF level and p-CREB/CREB ratio. These findings suggested that echinacoside may produce antidepressant-like behavioral effects in CUMS-induced depression by improving hippocampal neurogenesis *via* increasing BDNF signaling. Furthermore, the pro-neurogenic activity of M2 microglia might have some relationship with BDNF signaling pathway. it has been reported that IL4-driven microglia (M2 phenotype) in the hippocampus trigger BDNF-dependent neurogenesis responding to chronic stress, helping protect against depressive-like symptoms (Luo et al., 2017; Lu et al., 2020; Zhang et al., 2021). It also has been reported that NF-κB induced the expression of several pro-apoptotic and anti-apoptotic genes, including BDNF (Giacobbo et al., 2018). BDNF could also be down-regulated by exposure to IL-1β or TNF-α (Patterson, 2015; Xu et al., 2015). In the present research, the microglia could be induced into M1 or M2 phenotype by IFN-γ or IL-4, respectively. Furthermore, it is interesting to find that IL-4-stimulated microglia have a higher expression of BDNF than IFN-γ-stimulated microglia, suggesting that the alternative activation of microglia may have relationship with the BDNF signaling pathway. Results in the present study showed that N9 microglia cells can be polarized into M1 phenotype induced by LPS. In contrast, echinacoside group decreased the CD86/CD206 ratio, TNF-α and IL-1β levels, and increased the IL-4 and IL-10 levels, indicated that echinacoside could convert microglia from M1 phenotype to M2 phenotype. At the same time, echinacoside up-regulated the BDNF level. These results indicated that M2 microglia activation might be the upstream of BDNF-CREB signaling involvement in the antidepressant-like effects of echinacoside.

## 4 Conclusion

Echinacoside ameliorates depression-like behavior in CUMS mice, possibly through enhancing BDNF-CREB pathway and inhibiting neuroinflammation *via* regulating microglia M1/M2 polarization and JAK1/STAT3 pathway.

## 5 Materials and methods

### 5.1 CUMS procedure

Adult male C57BL/6 mice weighing 18–20 g were obtained from Beijing Vital River Laboratory Animal Technology Co., Ltd. The approval number for animal experiments is SCXK (Beijing) 2016–0006. All mice were housed in a 12 h dark/light cycle, in a temperature (18°C–22°C) and humidity controlled environment with unlimited access to water and food. The CUMS procedure was followed as described with some adjustment (Duan et al., 2020). Briefly, weekly stressors are listed as follows: 1) water or food deprivation for 12 h, 2) wet bedding for 24 h (200 mL osf water per cage), 3) cage tilting (45°) for 24 h, 4) cage shaking for 10 min, 5) swimming in cold water (4°C) for 5 min, 6) swimming in hot water (40°C) for 5 min, 7) overnight illumination for 12 h, 8) restraint stress for 2 h, (9) tail clipping for 1 min. A total of nine types of stimulation (more than one per day) were applied randomly to animals and various stressors were used on two consecutive days for 7 weeks, so that mice could not predict the occurrence of stimulation. The control animals were housed in a separate room and had no contact with the stressed animals, and mice that underwent CUMS treatment were kept in individual cages until the final behavioral test. This experiment was conducted by the Regulations on the Management of Laboratory Animals and the Guide for the Care and Use of Laboratory Animals of Henan University of Traditional Chinese Medicine, promulgated by the National Science and Technology Commission of the People’s Republic of China. The ethical approval reference number for this research is DWLL2018080003. All procedures for the care of mouse were following the institutional guide-lines for the use of animals in the study.

### 5.2 Drug treatment

**FIGURE 13:**
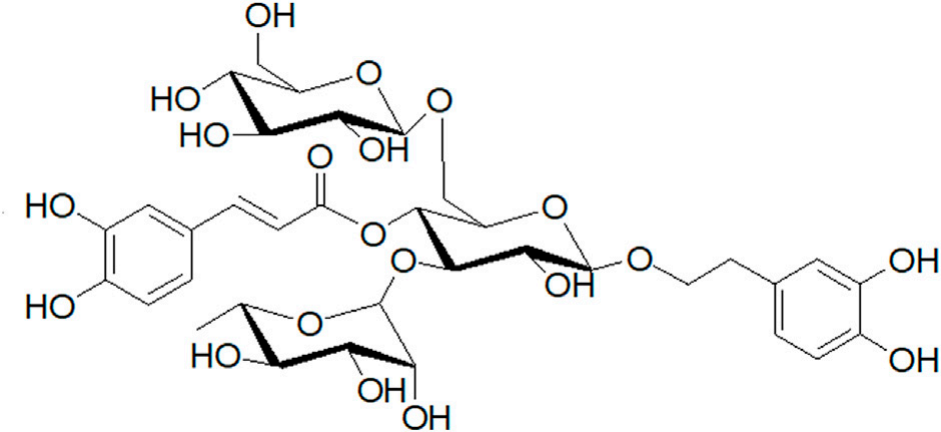
Structure of echinacoside.

After mice received the CUMS procedure for 5 weeks, they were randomly divided into five groups (6 animals in each group). NC: un-treated normal mice; CUMS: mice were treated with the chronic stimulating factors for 7 weeks to set up the CUMS depression model; FLX: CUMS induced mice were administered with fluoxetine (10 mg/kg,i.g.) daily for 21 days; ECH-L: CUMS induced mice were administered with echinacoside (15 mg/kg,i.g.) daily for 21 days; ECH-H: CUMS induced mice were administered with echinacoside (60 mg/kg,i.g.) daily for 21 days. The echinacoside was purchased from Chengdu Pufei De Biotech Co., Ltd., and the structure of the compound is shown in Figure 13. The doses and administration method were selected based on the previous studies (Motaghinejad et al., 2017; Chen et al., 2020). Fluoxetine was bought from PATHEON FRANCE.

### 5.3 Behavioral tests

**FIGURE 14:**
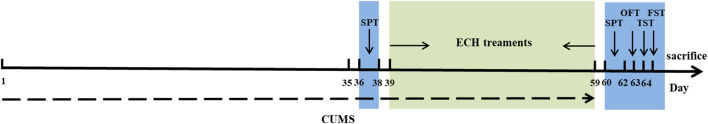
Schematic representation of timeline for echinacoside treatment in experimental animals with CUMS-induced depression.

All behavioral tests were used as indices to evaluate the effectiveness of the drugs on the depressed mice. After the CUMS procedure, sucrose preference test (SPT), open field test (OFT), tail suspension test (TST), and forced swimming test (FST) were performed in order with a 24 h interval between each test. The whole experimental procedure is shown in Figure 14.

#### 5.3.1 SPT

The test was performed as described previously with minor modifications (Liu et al., 2017). Briefly, each mouse was individually placed in a cage with 1% (w/v) sucrose solution in two bottles for 24 h, followed by one bottle filled with fresh water for another 24 h. After adaptation, mice were deprived of water and food (12 h) and then were free to access two bottles containing 100 mL of sucrose solution (1% w/v) and 100 mL of water. After 12 h, the weights of consumed sucrose solution and water were recorded and the sucrose preference was calculated as: sucrose preference (%) = sucrose consumption × 100% (sucrose consumption + water consumption).

#### 5.3.2 OFT

The OFT was designed as described previously (Cao et al., 2021). Each mouse was placed in the center of the white open-field box (60 × 60 × 60 cm) and allowed free access to explore the area for 5 min. The floor of the arena was divided equally into nine zones and supervised by a camera secured to the top of the apparatus. Total distances and immobility time were measured. After each trial, the apparatus was cleaned with 75% ethanol to eliminate any smell. This experiment was conducted using the OFT-100 experimental analysis system (Taimeng Software Co., Ltd., Chengdu, China).

#### 5.3.3 TST

The TST was used to assess behavioral despair, which is a feature of depression. The test was performed as described previously with minor modifications (Cao et al., 2021). Briefly, mice were suspended by adhesive tape placed approximately 1 cm from the tail-tip with the head 40 cm above the floor. Each animal was isolated to avoid interference during the experiment. The trial lasted for 5 min and the total test procedure of mouse immobility time was counted during a test period of 5 min (a mouse was considered to be immobile when it hung passively and motionless during the last 3 min of the test). Data was recorded by two separate operators.

#### 5.3.4 FST

The FST was conducted as described in the literature (Frenois et al., 2007). Each mouse was individually placed in a container (20 cm height, 10 cm diameter) with water at a height of 20 cm (25°C ± 1°C) for 5 min. The immobility time during the last 3 min was detected (floating with only small movement necessary to keep the head above water). Data was recorded by two separate operators.

### 5.4 Tissue sampling

At the end of the experiment, the animals were sacrificed after the behavioral tests. Brains were rapidly removed, and the entire hippocampus was rapidly isolated. The samples were stored at −80 °C until further estimations.

### 5.5 Histopathological observation by HE staining

Whole brain was removed and post-fixed immediately in 10% cold formalin for 1 day at 4°C, and then every tissue sample was dehydrated in alcohol gradient, cleaned in xylene, and embedded in paraffin. Successive brain coronal slices (5 μm thick) were made by microtome (Leica, Germany). Next, the sections were taken for deparaffinization and rehydration for histopathologic staining. Tissue specimens were stained with hematoxylin and eosin (HE), and histopathological changes of brain tissue were observed using light microscopy (XSP-C204, CIC, China) and images were acquired.

### 5.6 Nissl staining

The hippocampus paraffin sections were dewaxed to water, immersed for 5 min in Nissl staining solution, and washed quickly in distilled water, followed by ethanol dehydration, xylene transparency, and neutral gum sealing. Finally, the sections were cleared in pure xylene, and then mounted with neutral gum for microscopy (XSP-C204, CIC, China).

### 5.7 Analysis of JAK/STAT signaling pathway and iNOS expression by immunohistochemistry

The tissue immunofuorescence step was performed as described previously (Cao et al., 2021). Briefly, paraffin-embedded slices were quenched to inactivate endogenous peroxidase for 30 min using 3%H_2_O_2_; then, the non-specific binding sites were blocked using 3% BSA (bovine serum albumin) at room temperature for .5 h. The sections were incubated overnight at 4°C with STAT3 (1:200; Bioss, Beijing, China), p-STAT3 (1:200; Bioss, Beijing, China), iNOS (1:500; Bioss, Beijing, China), JAK1 (1:200; Affinity, OH, United States), p-JAK1 (1:200; Affinity, OH, United States). After washing, sections were incubated for 50 min in the appropriate secondary antibody at 37°C. Finally, sections were developed with 3,3′-diaminobenzidine (DAB) (G1211, Servicebio, Wuhan, China), and then the nuclei were counterstained with hematoxylin. A light microscope (XSP-C204; CIC, Chongqing, China) was used to observe the sections, and the intensity of the stained areas for each group was analyzed using Image-Pro Plus 6.0 software (Media Cybernetics, MD, United States) (3 pictures per animal).

### 5.8 Determination of HPA axis hormone, neurotransmitter and inflammatory factors by enzyme-linked immunosorbent assay (ELISA)

Blood samples were collected and centrifuged at 3000 rpm/min for 15 min at 4 °C. The supernatant was then placed in Eppendorf tubes and stored at –80°C prior to detection of the concentrations of CRH, ATCH, and CORT (Calvin Biotechnology Co., Ltd., Jiangsu, China) (the assay ranges are 2.5–80 pg/mL, 2.5–80 pg/mL and 7.5–240 ng/mL, respectively; the minimum detectable doses are typically less than 1.0 pg/mL, 1.0 pg/mL and 1.0 ng/mL, respectively). The hippocampus samples were homogenized with cold .9% saline and centrifuged at 5000 *g* for 10 min to collect supernatant, which was used to measure the content of 5-HT, NE, and DA (Elabscience Biotechnology Co., Ltd., Wuhan, China) (the assay ranges are 15.63–1000 ng/mL, .31–20 ng/mL and 31.25–2000 pg/mL, respectively; the minimum detectable doses are 9.38 ng/mL, .19 ng/mL and 18.75 pg/mL, respectively) and IL-1β, IL-6, TNF-α, and IDO (Calvin Biotechnology Co., Ltd., Jiangsu, China) (the assay ranges are 3.75–120 pg/mL, 3.75–120 pg/mL, 20–640 pg/mL and 1.5–48IU/mL, respectively; the minimum detectable doses are typically less than 1.0 pg/mL, 1.0 pg/mL, 1.0 pg/mL and 1.0I U/mL, respectively) with ELISA kits according to the manufacturer’s instructions. Each index concentration of sample was calculated from the linear equation derived from the standard curve of known concentrations of indicators.

### 5.9 Determination of Iba-1 level by immunofluorescence assay

After brain tissue sections were taken for deparaffinization, rehydration, antigen retrieval, endogenous peroxidase, and bovine serum albumin (BSA) block, sections were incubated with rabbit anti-Iba-1 overnight at 4°C (1:1000, Servicebio) for labeling the microglia in hippocampus. After being washed in phosphate-buffered saline (PBS) 3 times, sections were incubated with fluorescence tagged secondary Cy3 antibodies (1:500, donkey anti-rabbit, Servicebio) for 1 h at 37°C. Lastly, sections were incubated with 4’,6-Diamidino-2-phenylindole (DAPI) solution at room temperature for 10 min to stain nuclei, and images were collected by fluorescent microscopy (Nikon DS-U3, Japan).

### 5.10 Analysis of CREB/BDNF signaling pathway by western blot

The hippocampus samples were homogenized with tissue protein extraction reagent (Solarbio, Beijing, China) containing protease inhibitors, and centrifuged at 12,000 *g* for 30 min at 4°C, and the supernatant was collected. The protein concentration was determined with a BCA kit (Solarbio, Beijing, China) following the manufacturer’s instructions. The proteins were separated by 12% SDS-PAGE gel at 120 V and then transferred to polyvinylidene fluoride (PVDF) membranes. The membranes were blocked with 5% (w/v) BSA at room temperature for 2 h and then incubated with primary antibodies p-CREB (1:1,000, Abcam), CREB (1:1,000, Abcam), BDNF (1:1,000, Abcam), and GAPDH (1:1,000, Abclonal) overnight at 4°C. After incubation, the membranes were washed with PBST for 5 times and then incubated with anti-rabbit or anti-mouse IgG secondary antibody (1:1000) for 1 h in the dark. After washing, the bands were scanned using Li-COR Odyssey CLx system, and signal strength was quantified with Image Studio Version 5.2 (LI-COR, Lincoln, NE, United States).

### 5.11 Cell culture and drug treatment

The N9 murine microglial cell line was purchased from Otwo Biotech Ltd. (Shenzhen, China), and was maintained in dulbecco’s modified eagle medium (DMEM) (Gibco, NY, United States), supplemented with 10% fetal bovine serum (FBS), 100 U/mL penicillin/streptomycin in an incubator at 37°C containing 5% CO_2_.

N9 cells (2 × 10^4^) were plated into 96-well plates, 3 × 10^4^ were plated into 24-well plates and 4 × 10^4^ were plated into 6-well plates. Then incubated at 37°C in 5% CO_2_ overnight. After 24 h, the cells were divided into six groups: Control group was incubated with medium, LPS group was induced with 1 μg/ml LPS only, positive group was exposed to medium containing minocycline (Mino) and 1 μg/ml LPS, and ECH group was treated with medium containing 5, 10, and 20 μM of echinacoside and 1 μg/ml LPS. After 24 h of treatment, cells and supernatants were collected for MTT assay. After 24 h, the cells were divided into four groups: Control group was incubated with medium, LPS group was induced with 1 μg/ml LPS only, positive group was exposed to medium containing minocycline (Mino) and 1 μg/ml LPS, and ECH group was treated with medium containing 20 μM of echinacoside and 1 μg/mL LPS. After 24 h of treatment, cells and supernatants were collected for further study.

N9 cells (2 × 10^4^) were plated into 96-well plates and 4 × 10^4^ were plated into 6-well plates. Then incubated at 37°C in 5% CO_2_ overnight. After 24 h, the cells were divided into three groups: Control group was incubated with medium, IL-4 group was induced with medium containing 10, 20, 40 ng/mL IL-4, IFN-γgroup was treated with medium containing 12.5, 25, and 50 ng/mL IL-4. After 24 h of treatment, cells and supernatants were collected for Nitrite Assay. After 24 h, the cells were divided into three groups: Control group was incubated with medium, IL-4 group was induced with medium containing 40 ng/mL IL-4, IFN-γgroup was treated with medium containing 25 ng/mL IL-4. After 24 h of treatment, cells and supernatants were collected for further study.

### 5.12 Cell viability detection by methylthiazol tetrazolium (MTT) assay

MTT assay was used to measure cell viability in cultures. Cells were plated at a density of 4000 cells/mL in a 96-well cell culture plate (Corning, Denmark). After administration, 20 μl of .5 mg/ml of MTT (Solarbio, Beijing, China) was added to each well and cells were incubated at 37°C for 4 h. Following that, 150 μl of dimethyl sulfoxide (DMSO) per well was added to solubilize the formazan crystals in viable cells. Optical density (OD) in each well was quantified at 490 nm wavelength using an ELx808 Ultra Microplate Reader (BioTek, Winooski, VT, United States).

### 5.13 NO production level measurement by nitrite assay

Accumulation of nitrite (NO_2−_) in the culture media, an indicator of NO synthase activity, was measured by Griess reaction (Wu et al., 2007). N9 cells (2 × 10^5^ cells/mL in .5 ml of DMEM) were grown in triplicate in 24-well plates. After 24 h, the four groups of cells were incubated with pure medium, 1 μg/ml LPS, .1 μM Mino+1 μg/ml LPS, or 5, 10, and 20 μM echinacoside +1 μg/ml LPS for 24 h. Then, 50 μl culture supernatants were mixed with 50 μl Griess reagent (Solarbio, Beijing, China) at room temperature. After 5 min, the absorbance was determined at 540 nm using the ELx808 Ultra Microplate Reader (BioTek, Winooski, VT, United States).

### 5.14 Cell morphology and iba 1 observation by fluorescent microscopy

After N_9_ cells were seeded in 96-well culture plate and treated, N_9_ microglia morphology was observed using high-content screening (Operetta, PerkinElmer, United States). Then, N_9_ was fixed with 4% paraformaldehyde in PBS for 15 min and permeabilized with .25% Triton X-100 for 10 min. Non-specific binding was blocked by incubating the cells in PBST containing 1% BSA for 30 min. Cell were incubated with rabbit-anti-Iba 1 (1:200) overnight at 4°C. After three washings, the cells were incubated with goat anti-rabbit IgG H&L (Alexa Fluor^®^ 488, 1: 500) in the dark for 2 h and the nuclei were stained with DAPI for 5 min. Fluorescent images were obtained using high-content screening.

### 5.15 Polarization markers imaging by flow cytometry

CD11b, CD86 and CD206 of N9 cell surface markers were detected by imaging flow cytometry. Briefly, cells were harvested by trypsin (without EDTA), then centrifuged at 1000 rpm for 5 min, and washed twice with cold PBS, and the cell pellet was collected. The cells were resuspended in 100 μL of PBS, then stained with CD11b-percpcy5.5 (1:80, eBioscience), CD206-PE (1:160, eBioscience), and CD86-APC (1:300, eBioscience) antibodies for 20 min at 4 C in the dark. All samples were detected using imaging flow cytometry (Image Stream X Mark II, Merck) and analysis was done with IDEAS 6.2 software.

### 5.16 Measurements of inflammatory factors and anti-inflammatory factors by ELISA

The levels of secreted IL-1β, IL-6, and TNF-α and IL-4, IL-10, and TGF-β in culture supernatants were determined by ELISA. N9 cells (2 × 10^5^ cells/ml in .5 ml of DMEM) were grown in triplicate in 24-well plates overnight, followed by incubation in the absence or presence of echinacoside. The culture supernatants were collected after 24 h of treatment, and the amount of IL-1β, IL-6, and TNF-α and IL-4, IL-10, and TGF-β release was determined using the respective ELISA kits (Calvin Biotechnology Co., Ltd., Jiangsu, China) following the manufacturer’s protocol.

### 5.17 Statistical analysis

Data were expressed as mean ± standard error of the mean (SEM) and analyzed by one-way analysis of variance (ANOVA) using SPSS 26.0 software (SPSS Inc., Chicago, IL, United States), with *p* < .05 considered as statistically significant.

## Data Availability

The original contributions presented in the study are included in the article/Supplementary Material, further inquiries can be directed to the corresponding authors.
